# Supplementary material for: Human multiethnic radiogenomics reveals low-abundancy microRNA signature in plasma-derived extracellular vesicles for early diagnosis and molecular subtyping of pancreatic cancer
Source: eLife. 2025 Aug 8;14:RP103737. doi: 10.7554/eLife.103737 (PMC12334162; doi:10.7554/eLife.103737)
Supplement: Figure 2—source data 1. [file elife-103737-fig2-data1.docx]

| **Figure 2C-source data 1 Important radiomic features** | |
| --- | --- |
| Id | Detail inforamtion of features |
| f_1_ | wavelet.HHH_ngtdm_Contrast |
| f_2_ | wavelet.HHH_glcm_SumAverage |
| f_3_ | wavelet.HHH_glcm_JointAverage |
| f_4_ | wavelet.LLL_firstorder_Kurtosis |
| f_5_ | wavelet.HLH_firstorder_Uniformity |
| f_6_ | wavelet.HHH_firstorder_Minimum |
| f_7_ | wavelet.HLH_firstorder_Entropy |
| f_8_ | wavelet.LLH_firstorder_Mean |
| f_9_ | wavelet.LLH_firstorder_Median |
| f_10_ | wavelet.HLL_firstorder_Median |
| f_11_ | log.sigma.1.mm.3D_glszm_GrayLevelNonUniformity |
| f_12_ | wavelet.HLH_firstorder_10Percentile |
