# Supplementary material for: Human multiethnic radiogenomics reveals low-abundancy microRNA signature in plasma-derived extracellular vesicles for early diagnosis and molecular subtyping of pancreatic cancer
Source: eLife. 2025 Aug 8;14:RP103737. doi: 10.7554/eLife.103737 (PMC12334162; doi:10.7554/eLife.103737)
Supplement: Figure 5—source data 1. [file elife-103737-fig5-data1.docx]

| **Figure 5A- source data 1** Shared targets of three EV miRNAs | | |
| --- | --- | --- |
| **hsa-miR-1260b** | **hsa-miR-151a-3p** | **hsa-miR-5695** |
| Candidate Targets | | |
| AATF,AZIN2,CABIN1,CECR2,DENND4B,EIF3B,EMB,FKBP15,GLMN,HADH,IKBKB,MLH3,MRPS21,NDE1,PIM3,PLEKHA2,PSMF1,PUM1,RNF125,SAE1,SAP30BP,SLC6A6,TCOF1,USP36,YWHAQ,ASCL5,CCDC140,CHEK2,DAPK2,FAM71F2,GIGYF2,HEATR5A,HIST1H2BJ,MPHOSPH9,NFASC,PCBD2,PRR13,SGK3,SH3PXD2A,SLC22A3,STON2,TOMM40,ZNF398,ZNF791,ABI2,ACAT1,ALDOA,APLP2,ATAD1,AUTS8,C16orf58,C2orf48,CCT7,CDCA4,CDKN1A,CKB,CLCN2,COL4A3BP,COPA,CTPS1,DDX3Y,DHX30,DNAH10OS,DNMT1,EEF1A1,EIF4G1,ENO1,EPHB4,EZH2,GAN,HIPK1,HNRNPA2B1,HNRNPA3,IKZF2,LGALS3BP,MCM7,MDN1,MKNK2,MRPL24,MRPL37,MYO5C,NBPF15,NCAPG2,NFE2L1,NOL8,OGG1,PALM2,PARP4,PLOD2,POLR2A,POLR3D,PPIAL4A,PRC1,PRKRIP1,PTPRF,RAB19,RAB5IF,RBMXL1,RPE,RPL3,RPL36A,RPL4,RPS15,RPS6,RPS7,SEC61G,SETDB1,SF3B1,SLC25A3,SLC39A8,SLC3A2,SLC7A5,SOGA3,SP3,TMEM59,USP12,WDR62 | | |
