# Supplementary figures and images for: Human multiethnic radiogenomics reveals low-abundancy microRNA signature in plasma-derived extracellular vesicles for early diagnosis and molecular subtyping of pancreatic cancer
Source: eLife. 2025 Aug 8;14:RP103737. doi: 10.7554/eLife.103737 (PMC12334162; doi:10.7554/eLife.103737)

# NMF rank survey

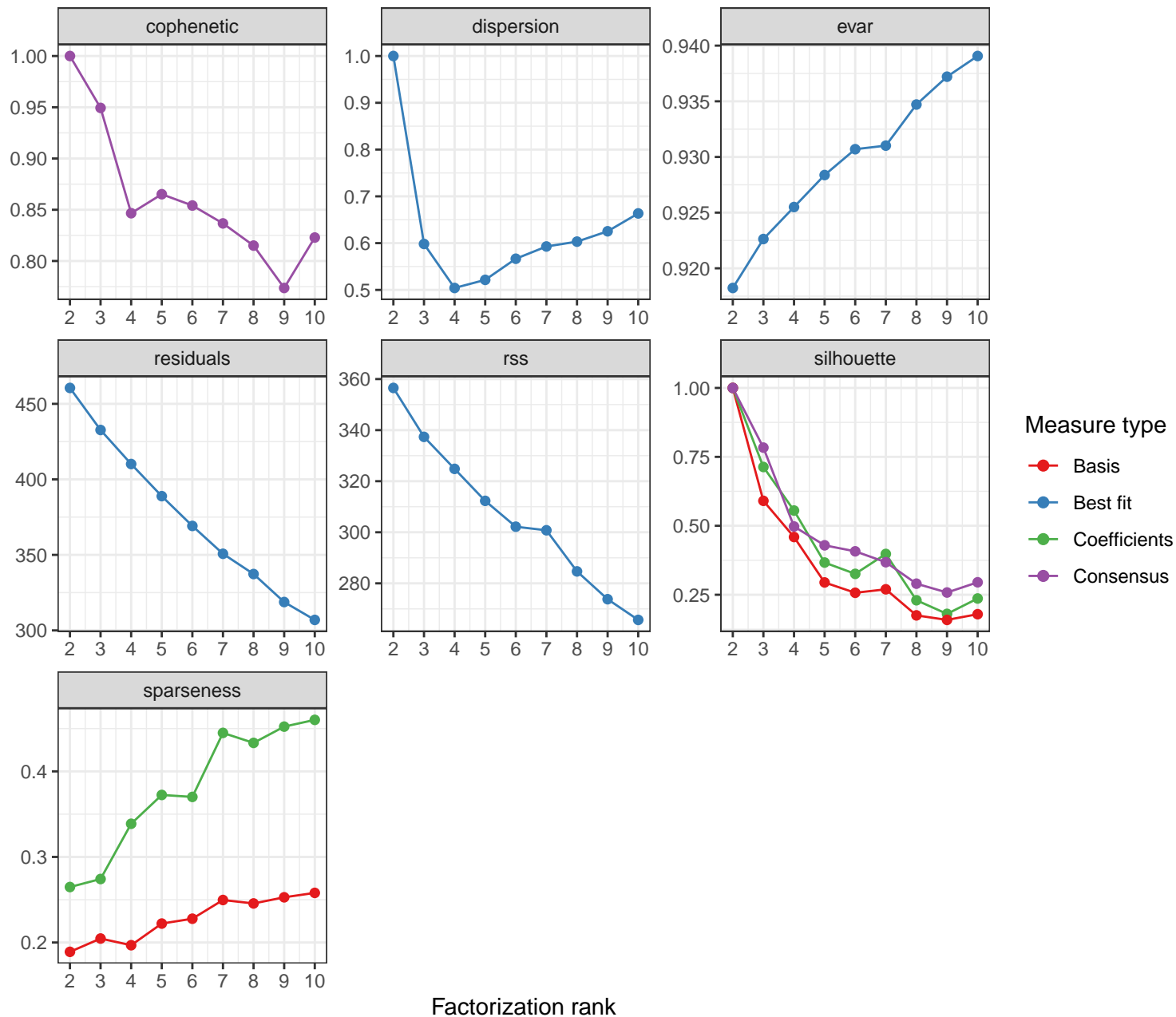

Supplement: Figure 5—source data 2. [file elife-103737-fig5-data2.pdf]
